# Successfully initiating an escalation of care in acute ward settings—A qualitative observational study

**DOI:** 10.1111/jan.16248

**Published:** 2024-06-27

**Authors:** J. Ede, B. Kent, P. Watkinson, R. Endacott

**Affiliations:** ^1^ Oxford University Hospital NHS Foundation Trust Oxford UK; ^2^ School of Nursing and Midwifery University of Plymouth Plymouth UK; ^3^ Nuffield Department of Clinical Neurosciences University of Oxford Oxford UK; ^4^ National Institute for Health and Care Research, Minerva House London UK

**Keywords:** acute care, adult nursing, critical care, ethnography, evidence‐based practice

## Abstract

**Aims:**

To address knowledge gaps by (i) developing a theoretical understanding of escalation and (ii) identifying escalation success factors.

**Design:**

Non‐participant observations were used to examine deteriorating patient escalation events.

**Methods:**

Escalation event data were collected by a researcher who shadowed clinical staff, between February 16th 2021 and March 17th 2022 from two National Health Service Trusts. Events were analysed using Framework Analysis. Escalation tasks were mapped using a Hierarchical Task Analysis diagram and data presented as percentages, frequency and 95% CI.

**Results:**

A total of 38 observation sessions were conducted, totaling 105 h, during which 151 escalation events were captured. Half of these were not early warning score‐initiated and resulted from bleeding, infection, or chest pain. Four communication phenotypes were observed in the escalation events. The most common was Outcome Focused Escalation, where the referrer expected specific outcomes like blood cultures or antibiotic prescriptions. Informative Escalations were often used when a triggering patient's condition was of low clinical concern and ranked as the second most frequent escalation communication type. General Concern Escalations occurred when the referrer did not have predetermined expectations. Spontaneous Interaction Escalations were the least frequently observed, occurring opportunistically in communal workspaces.

**Conclusion:**

Half of the events were non‐triggering escalations and understanding these can inform the design of systems to support staff better to undertake them. Escalation is not homogenous and differing escalation communication phenotypes exist. Informative Escalations represent an organizational requirement to report triggering warning scores and a targeted reduction of these may be organizationally advantageous. Increasing the frequency of Spontaneous Escalations, through hospital designs, may also be beneficial.

**Impact Statement:**

Our work highlights that a significant proportion of escalation workload occurs without a triggering early warning score and there is scope to better support these with designed systems. Further examination of reducing Informative and increasing Spontaneous Escalations is also warranted.

**Patient and Public Contribution:**

Extensive PPIE was completed throughout the lifecycle of this study. PPIE members validated the research questions and overarching aims of the overall study. PPIE members contributed to the design of the study reviewed documents and the final data generated.

## 1 INTRODUCTION

Improving care for the deteriorating ward patient is a National Health Service (NHS) (Hogan et al., 2019) and international priority. In‐patient deterioration can result from physiological or biochemical instability (Mohammed Iddrisu et al., 2018). To avoid worsening instability, an escalation of care is required whereby clinical staff recognize and communicate this deterioration to specialist teams and implement first line treatments (Johnston et al., 2016). Failure to escalate has been cited to be between 10%–50% and can result in cardiac arrests, unplanned intensive care unit (ICU) admissions (Hogan et al., 2019) and increased ICU mortality and morbidity rates (Magor et al., 2022; McQuillan et al., 1998; Stelfox et al., 2014). Up to 1% of ICU admissions may be avoided with timely and appropriate care (Redfern et al., 2020).

### 1.1 Background

The two main escalation processes are an Afferent (recognition and communication of deterioration) and Efferent limb (management of patient deterioration) (Odell, 2015). Early Warning Score (EWS) systems aim to improve the Afferent limb by facilitating healthcare staff to recognize deterioration and signpost clinical actions (increasing the frequency of monitoring or further support) (Hogan et al., 2019). However, there is some evidence to suggest that a number of patients are transferred to the ICU without triggering an alert (Nestor et al., 2022). Similarly, clinical staff frequently cite examples when patients do not meet the required EWS threshold but are clinically concerning (Ede & Endacott, 2021). Some warning systems account for this and have successfully balanced mandated responses with clinical judgement (Pain et al., 2017). The escalation workload related to patients who do not meet the EWS thresholds is limited predominantly because they are difficult to identify through traditional hospital systems (Ede et al., 2021).

The concept of escalation is described homogenously and lacks nuance within the literature. Escalation communication, which adequately relays patient risk across healthcare teams, remains central to patient safety (Bradley et al., 2015) but is often described in transactional terms. Communicating risk during deterioration dialogues is multifaceted, challenging, can result in a risk mismatch between parties (Lavoie et al., 2020) and the use of a standardized process (such as SBAR) does not eliminate all communication failure. Evidence suggests that when surveyed about the choice of using an online referral system or a verbal interaction, clinical staff preferred a conversation (Amarouche et al., 2017). This infers that escalation is more than a transaction of information; the output of which evolves because of the verbal discussion. Seminal work on deterioration events indicates a greater understanding of communication is central to informing further process improvements (Ghaferi & Dimick, 2017).

Overall, there is a lack of detailed evidence fully describing escalation in the non‐triggering patient and there appears to be an assumption of escalation communication homogeneity. The aims of this research are to address these gaps by (i) developing a theoretical understanding of escalation and (ii) identifying escalation success factors. Objectives were to:

- Observe escalation events in the acute ward setting of medical, surgical and trauma patients
- To report the process of escalation
- To report escalation success factors derived from observations

## 2 DESIGN

This manuscript reports one phase of a wider mixed‐methods, multi‐site study (SUFFICE) examining escalation of care in the deteriorating ward patient. A full description of the methods informing the SUFFICE study may be found in the published protocol (Ede et al., 2021). This study was registered with the International Standard Randomized Controlled Trial Number organization (study number: ISRCTN 38850) and this manuscript has been reported against the COREQ checklist (see File S1). An observational approach was chosen to understand the contextual environment and collaborative process of escalation, and has been used as a method to collect data in other deterioration studies (Chua et al., 2013; Johnston et al., 2014). In order to minimize the Hawthorne Effect (McCambridge et al., 2014), non‐participant observations were utilized where the observer did not directly influence the phenomena of interest (Handley et al., 2020). Escalation of care is broadly defined as any communication relating to the recognition of patient deterioration (Johnston, 2015) or clinical change. A success factor was defined as any mechanism, context, or process that promoted a completed escalation of care, and this included the recognition and communication of clinical concerns across professional groups.

### 2.1 Sample

Data on escalation events for medical, surgical, and trauma patients were collected from two NHS Trusts from February 16th, 2021 to March 17th, 2022. Various clinical staff, including consultants, senior and junior doctors, sepsis specialist nurses, outreach practitioners, practice development nurses, and ward coordinators, were purposefully observed. This aimed to capture their interactions with ward staff during escalation events. Previous observations revealed challenges in timing when shadowing ward staff and being in the ‘right place at the right time’ to capture an escalation event. Thus, purposive sampling focused on maximizing proximity to escalation events to observe nurse interactions effectively. Ward staff were then asked to clarify what triggered them to escalate, what were their clinical concerns and what their expectations were of the escalation event was. Observations were conducted across entire hospital sites (see File S2 for observed clinical area descriptions) rather than being limited to single wards, depending on the individuals being shadowed and the locations of unwell patients.

### 2.2 Data collection

#### 2.2.1 Observational data

Escalation event observation data included both quantitative data (patient age, trigger score) and qualitative data (field notes, ad hoc question responses). Pre‐defined variables were developed during supervision sessions, through previous escalation of care ethnography work and drawn from the Qualitative Evidence Synthesis (Ede et al., 2021) informing the study. The electronic case report form (e‐CRF) tool was specifically developed in an Excel spreadsheet (Microsoft Corporation, 2018. Microsoft Excel, Available at: https://office.microsoft.com/excel). It was user tested prior to the formal data collection process and iterated by adding some quick drop‐down menus, categorizing certain anticipated qualitative data e.g., NtN (Nurse to Nurse) referral, NtD (Nurse to Doctor), and removing extraneous information. Scores that contributed to a larger score such as EWS (breakdown by parameter) were collected at an individual level whenever possible. Data inputted into the e‐CRF were anonymised at the point of capture.

Observations of escalation events focused on the interactions between clinical staff and other staff groups, to capture the collaborative and multi‐professional nature of the process. Data were collected by one researcher (JE) trained in ethnographic methods. The observer was not previously known to the participants and was not clinically affiliated with the areas under observation. No direct patient observations, identifiable patient/staff data were collected. Sessions were limited to a maximum duration of 4 h and staff members were observed at multiple times at various shift time‐points (early, late, night, and day) across different month clusters to capture any temporal or seasonal variations. Data (field notes, researcher reflections/memoirs, interview data) were collected with an e‐CRF which was developed by the research team and piloted.

#### 2.2.2 Ad hoc interview data

To document staffing, specific events or behaviours, field notes and observations were supplemented with ad hoc interviews. Staff were also asked to clarify their trigger for escalating, their clinical concerns, and a narrative surrounding the patient. These were short discussions with staff lasting no longer than 20 min.

### 2.3 Ethical considerations

Permission to conduct this research was granted by the Queens Square Research Ethics Committee (REC) reference number HRA‐20HRA/3828 and both hospital research departments. During this study, two consenting processes were employed to reduce the inadvertent observation of someone who may not have directly consented. Given the collaborative nature of escalation and the fluid nature of the observation sessions across multiple clinical environments, it was not possible to consent all observed clinical staff prior to observations. In the first instance, clinical staff who were directly shadowed provided written consent before the observation session commenced. Clinical staff that were indirectly observed (due to the nature of deteriorating patient management and care delivery in the acute ward) were asked to provide verbal agreement to being observed on initial contact so as not to interrupt the clinical workflow when managing a deteriorating patient. This was done out of professional courtesy and ensured that staff felt empowered to stop the observations. Retrospective consent was obtained once the observation or escalation event had concluded.

Staff were assured that observations were not focused on critiquing medical or nursing care but aimed at understanding the collaborative process of rescue. Before the start of the study, the divisional matrons and lead consultants were contacted and informed about the goals and objectives of the research. Ward managers were provided with an email to notify staff of the possibility of being observed, and how to object to observations. Researcher safety was paramount due to the onset of COVID‐19 infection and adherence to hospital and Public Health England (PHE) advice on PPE was required during observation sessions.

### 2.4 Data analysis

Data were inputted directly into a spreadsheet during and following the observations. Hand drawn diagrams were copied and refined in PowerPoint. Data analysis was completed as follows:

- Quantitative escalation event data were checked for errors and cleaned. Data are presented as proportions (%, 95% CI). Confidence intervals of proportions were calculated using the Clopper–Pearson method.
- Qualitative data from observations in field notes were read several times to allow the researcher to become familiar with the content.
- Tasks were documented by process mapping (Lane et al., 2006) within a Hierarchical Tasks Analysis (HTA). Hand drawn and sketched HTA drawings were refined based on the content of the qualitative fieldnotes data and researcher reflections. The HTA provides a theoretical framework for understanding the escalation process and serves as a basis for analysing the qualitative data.
- Qualitative data were summarized in a Framework Analysis matrix with the specific aim of identifying escalation success to the main sub tasks identified within the HTA. Coding was developed through team consensus in data meetings.
- The theory of Escalation phenotypes was tested across multiple observation sets and definitions were refined. Whilst escalation types have been defined and categorized, there is some overlap between them.

It is unlikely that saturation of themes would occur when examining the phenomenon of escalation. For this reason, the data's information power was considered and 151 escalation events, whilst not exhaustive, allowed the researchers to fulfil the aims of the study (Malterud et al., 2016).

### 2.5 Rigour

Comprehensive field notes were documented throughout and after the observation sessions. Notes consisted of direct observations (descriptions of tasks), direct staff quotes from ad hoc discussions (centred on the escalation event), researcher conceptual diagrams (HTA) and researcher reflections/memoirs. The observation data collection tool was trialled and refined as the sessions continued, which included creating some categories of commonly observed events (e.g., face to face referrals abbreviated to F2F). All observations were completed by one researcher (JE) who has a critical care background and acute ward experience. JE had previous training on qualitative research methods including techniques of ethnography and had conducted observation work in previous research related activities.

## 3 RESULTS

A total of 38 observation sessions were conducted at different standard shift time points, including early shift 30/38 (79%), late shift 6/38 (15.8%), and night shift 2/38 (5.2%), resulting in a cumulative observation duration of 105 h. Several clinical staff were shadowed including consultants, senior doctors, junior doctors, sepsis specialist nurses, outreach practitioners, practice development nurses, and ward co‐ordinators. A breadth of ward processes was also observed which included ward safety huddles, ward rounds, shift or team handovers, acute admissions, and ‘Hospital at Night’ meetings.

### 3.1 Escalation events

**FIGURE 1:**
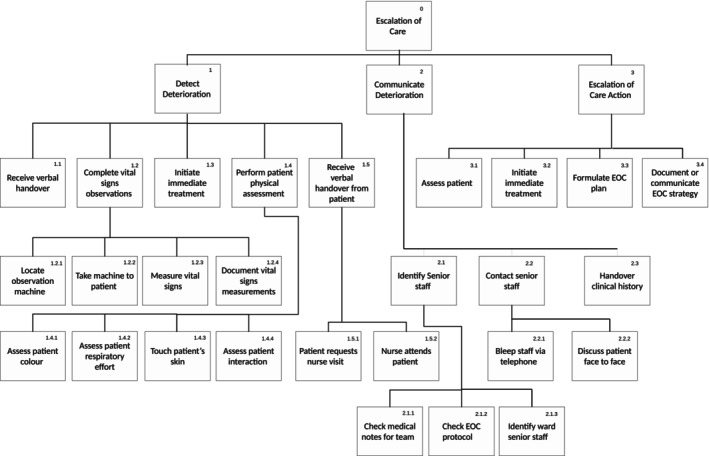
Hierarchical task analysis (HTA) of escalation of care.

A total of 151 escalation of care events were captured for patients in the following clinical specialities: medical 81/151 (53.6%), surgical 65/151 (43.0%), trauma 1/151 (0.7%), and unknown 4/151 (2.6%). Of these, 66/151 (43.7%) were female and 10 events had missing gender data as no direct patient observations were conducted. Key escalation steps observed were documented using a Hierarchical Task Analysis (HTA) (see Figure 1).

The HTA consists of three top level escalation sub tasks (detection of deterioration, communication of deterioration and escalation of care action) and 27 sub‐level tasks. Detecting deterioration required the completion of the highest number of lower‐level sub‐tasks (*n* = 15). Communicating deterioration (*n* = 8) and escalation action (*n* = 4) had fewer lower‐level sub‐tasks.

#### 3.1.1 Detection of deterioration (sub task 1)

Among escalated patients, the majority had an EWS of 3 or lower 66/151 (43.7%). The number of observed events decreased with increasing EWS scores: EWS 4–7, 53/151 (35%), EWS 8–11, 26/151, (17.2%), EWS >12, 3/151 (1.9%), and 3/151 (1.9%) events had missing EWS data (see File S3; Figure 1 for EWS score frequencies and distribution). Half of the escalations 77/151 (51%) were not initiated through concern surrounding the patient's EWS score (Non‐EWS initiated) escalation versus EWS initiated escalations 74/151 (49%) (see File S3; Figures S2, S3). This was also supported by the Qualitative data ‘*Twice daily assessment of hospital‐wide NEWS scores. We do have data to show that most of our referrals are based around nurse concern*.’ Observation Sessions 8, outreach nurse 2. The commonest clinical concerns for EWS‐initiated escalations were sepsis 11/74 (14.8%), hypotension 10/74 (13.5%), low Glasgow Coma Scale (GCS) 7/74, (9.5%), and hypoxia 6/74 (8.1%). The commonest clinical concerns for Non‐EWS initiated escalation were bleeding 7/77 (9%), infection 4/77 (5.2%), chest pain 4/77 (5.2%), and resolved desaturation 4/77 (5.2%) (see S3; Table S1,S2).

Generally, the detection of ward patient deterioration came from the assessment of vital signs, patient complaints, nursing assessments, automated alerts, or team handovers (see File S4). Deterioration detection was completed by nurses or medical staff, but other actors of escalation were captured such as healthcare support workers, student nurses and housekeepers. Staff also gave examples whereby family members of patients had recognized pending deterioration earlier than the clinical staff or re‐escalated unresolved concerns to outreach, which resulted in a critical care admission. Organizational visibility of deterioration improved clinical staff, ward managers, outreach, and medical teams' awareness of unwell ward patients allowing them to maximize the clinical support they could provide. This was generally achieved through electronic EWS, or laboratory results presented via interfaces such as whiteboards or mobile devices. Increased visibility also meant that some staff (outreach, sepsis nurses) had the ability to proactively identify unwell patients before an official escalation was initiated. To ensure organizational visibility of those patients who were clinically concerning but not triggering, one Trust was trialling the use of a Nurse Concern criteria along with EWS.

Staff described a complexity to deterioration detection, giving examples where some diagnosis criteria were not met once first line treatments were given such as fluids and Oxygen in septic patients. Similarly, staff often commented that escalating a patient with a raised EWS was easier for more junior staff. There were instances where detection of deterioration was done in the absence or before clear objective indicators (rising blood counts in the absence of fever or poor progression). This added further difficulty to sense making, and in some cases the ability to convincingly convey risk to other teams required for that's patients care. Conversely, there were examples where clinical staff were confident in their ability to anticipate or predict deterioration and created positive workarounds based on this. For instance, they adapted technology (mobile devices) to generate specific alerts relating to the patients' blood results day 5 following surgery, as this was when their patients were most likely to deteriorate.

#### 3.1.2 Communication of escalation (sub task 2)

Communication of escalation events occurred mostly between a nurse and a doctor, nurse to nurse or doctor to doctor through mobile devices, bleeps, team handovers or safety huddles. Communicating escalation proved to be challenging at times due to environmental factors such as ward configuration, large geographical areas, or front door patient access. Organizational factors could compound escalation challenges, such as multiple medical teams being responsible for patients which resulted in one nurse manager having 38 patients with 9 consultants leading care on a single shift. This posed a significant number of issues when trying to identify which medical team to escalate to and created a time‐consuming escalation process (see File S4).

Social interaction played a role during escalation communications and was particularly evident in escalations involving the outreach team and static medical consultants who were well acquainted with the acute ward staff. For example, outreach weighted medical information differently depending on context such as the ward's familiarity with unwell patients. The importance of communicating concern efficiently and creating the correct deterioration narrative was frequently raised by clinical staff so their patient was suitably prioritized for a response. Clinical staff had adapted the way escalation was communicated depending on the patient context (success factor) and their requirements of that interaction. Four escalation phenotypes were subsequently identified across the escalation events; Outcome Focused Escalation, Informative Escalation, General Concern Escalation, and Spontaneous Interaction Escalation attributes are described in the following section and have been summarized in Table 1. There were 137/151 escalation events captured in which the researcher was able to identify the escalation type (see File S5 for SPSS 95% CI outputs). Table 1 Definitions of Escalation of Care Phenotypes.

**TABLE 1:** Definitions of escalation of care phenotypes.

Escalation phenotype	Key attributes	Excerpt from field notes/ad hoc interviews/researcher reflections
Outcome focused escalation	Most common phenotype of escalation Outcome was pre‐anticipated by referrer Often preceded by a full patient review and strong clinical reasoning Efficiently prioritized	‘Nurse describes a patient escalation that she had last week. She knew the patient was unwell and felt the medics were slower to take control. She escalated up to the reg who agreed… Nurse knew the patient needed an intervention and conservative management would not reverse deterioration alone. She escalated knowing what she needed’ Site A/Nurse 2 Nurse escalated to team. Patient has been deteriorating overnight and had initial dose of digoxin. She was very firm in asking for an urgent review ‘I don't want this patient to deteriorate further’. Site A/ Esc 43 ‘Really required an escalation plan as patient 90. Patient clearly very unwell, high trigger score. Being treated for sepsis. Newly admitted so yesterday unlikely to be able to limit care or initiate palliative care pathway.’ Site A/ Esc 45 ‘If you don't use the right language to escalate then it may not be taken seriously’ Previous call today, complete jumble. Advised to use SBAR to organize call’ Site B/Observation Session 6
Informative escalation	Frequently observed To fulfil organizational requirement Generally, has about a low clinical concern Usually does not require a medical review May be a ‘reverse escalation’ to avoid the automatic escalation of flagged patients (False Positive)	‘Sometimes staff will escalate just because of a score but should document if not escalating.’ Site B/ outreach nurse ‘Referral to outreach can sometimes be a way to shed responsibility.’ Observation Session 10 ‘Just letting you know as the patient is triggering. outreach ask if they need any fluid prescribing. Patient is probably going to be palliated…..’ Site B/Esc 26 ‘Staff aware that patient was reaching end of life care. Escalation was informative to just let you know. This was to ensure that there was an awareness of treatment direction for the day team.’ Site B/ Esc 42 ‘Patient on incorrect NEWS2 scale and therefore triggering so the ward was notifying that the system EWS was incorrect.’ Site B/ Esc 58
General concern escalation	Not employed frequently No clear outcome requirement from referral Related to softer signals of patient deterioration	‘HCA escalated to ward round due to patient complaint and sweaty. Noted to be short of breath on exertion and unable to wean oxygen. ‘Site A/ Observation Session 1 ‘We recently had a patient that was referred to us by their family, who became progressively more unwell and was admitted to ICU. We have had several examples where patients care has been directly altered due to a family escalation to outreach’ Site B/outreach nurse 1 ‘Housekeeper escalated patient complaint of pain to the nurse in charge.’ Site A/ Esc 28 ‘During one session I was shadowing a surgical ward round. The MDT were reviewing a patient within the side room. During this time an HCA came out of the opposing side rooms and spoke to the nurse in charge. I found out that the HCA had just been mobilizing this patient with the physiotherapy team which she had done previously. She was concerned because the patient was notably short of breath of exertion, more so than previous rehabilitation sessions. The nurse in charge suggested that this patient been seen next and diverted the ward round to this patient. This started several interventions such as a chest x‐ray and full medical review’ Site A/Observation Session 3
Spontaneous interaction escalation	Least common type of escalation phenotype Occurred during informal discussions or in joint clinical workspaces ‘Opportunistic in nature’ Heavily influenced by workspaces creating ‘discussion zones’ Form of social interaction Driven by organizational awareness of unwell patients Prompted by alerts, whiteboards, or mobile devices	‘Whilst observing Site B's Sepsis Nurse a concurrent escalation was observed. Patient A was unwell on ward XX and was alerting for sepsis. The Sepsis Nurse specialist begins her day by assessing all automated sepsis alerts and remotely reviews each patient. Patient A had alerted for increased NEWS2 signals and laboratory results indicating a severe infection. She shares the same office as the ICU outreach team so proceeds to refer Patient A prior to going to see the patient. A few minutes after the sepsis nurse's verbal handover, the ICU outreach Team received a bleep from the ward nurse caring for Patient A to refer him due to NEWS >7 and sepsis alerts. Reflecting on this there are many mechanisms at play. Technology features heavily in this escalation event which allowed staff to have knowledge of the unwell patient prior to any referral being made. Having teams which ‘seek out the sick’ appears advantageous.’ Site B/Observation Session 20 ‘Senior nurse reviewed dashboard and interrogated notes due to high trigger and sepsis flag on dashboard (not their patient). Initiated a discussion with doc to ask about antibiotics…This flag is generated from observations ‘Site A/ Esc 17. ‘Outreach was concerned by a nurse's tone of voice (appeared unnerved), so they (outreach) decided to visit ward regardless although unlikely to add much to patient's care. Known that this ward do not usually have very sick patients and therefore may need some support’ Site B/Observation Session 9 ‘Outreach decided to proactively review patient with the ENT team (who was just reviewing the patient) just in case they were asked to review again overnight, and they could handover a full clinical picture to the night outreach cover.’ Site B/Esc 30 ‘Systems that seek deterioration seem to find it. The outreach team actively review all the EWS throughout the hospital and rank them according to acuity’ Site B/Observation Session 7 ‘Once a shift we see a patient who is triggering but not been referred…we find them when reviewing Trust‐wide EWS scores. This may be because they are chronic high NEWS, palliative or known to team.’ Site B/ outreach nurse 2

#### 3.1.3 Output focused escalation

Output Focused Escalation was the most common accounting for 57/137 escalations (41.6%, 95% CI 33.3–50.3). Staff often anticipated the required output of escalation (i.e., what was required to manage the patient clinical deterioration or further diagnostic investigations) such as blood cultures, fluid boluses or medical review and this was communicated, or suggested. Output Focused Escalation was followed by a highly structured patient assessment by the bedside nurse, which contained multiple data points to support clinical suggestions and demonstrated a convincing referral when bidding for clinical time. These data points may have been generated from EWS, other signals of deterioration or patient/relative/other staff concern. Staff indicated that this was a more effective type of escalation when critical actions were required. In some instances, this escalation was employed to initiate end of life discussions when patients were becoming more unstable and at risk of unnecessary interventions.

#### 3.1.4 Informative escalation

Informative Escalation was the second most frequently observed escalation type accounting for 49/137 events (35.8%, 95% CI 27.8–44.4). This approach was most likely to be employed in cases where a patient's EWS score indicated a need for further assessment or intervention, but the level of clinical concern was relatively low. The communication episode was often employed to fulfil an organizational or a local escalation policy requirement and to ensure due diligence, but often had little clinical effect. A medical review was generally not required, and the communication content consisted of ‘just to let you know’. Informative Escalations also consisted of ‘reverse escalations’, where patients were flagged electronically (False Positive) but this alert needed to be overridden following a clinical judgement, and an escalation actively avoided (patient on the palliative care pathway). The NEWS2 scale generated the need for ‘reverse escalations’ due to patients being on the wrong oxygen scale and falsely triggering.

#### 3.1.5 General concern escalation

General Concern Escalation was employed much less frequently and evident in just 26/137 escalation events (19%, 95% CI 12.8–26.6). These escalations related to patients with no clear signs of deterioration such as poor weaning of oxygen, confusion, or mobility changes. The referrer did not state any preconceived ideas about what the cause of the clinical concern was or the required outcome of the escalation. This was often based on a ‘gut feeling’ of deterioration and lacked structured evidence from EWS, or assessment of other data points.

#### 3.1.6 Spontaneous interaction escalation

Spontaneous Interaction Escalation was the least frequent, being observed in 5/137 (3.6%, 95% CI 1.2–8.3) events. These were informal face‐to‐face discussions occurring in joint clinical workspaces and were a type of ‘social interaction’. The ease at which these escalations occurred was influenced by the team structure and socio‐cultural factors. Some Spontaneous Interaction Escalations were driven by organisational awareness of deterioration through electronic vital signs alerts, whiteboards, or mobile devices. Some teams (sepsis, outreach) were seeking out unwell patients through deterioration surveillance and these interactions may have preceded a formal referral.

#### 3.1.7 Escalation action (sub task 3)

Actions surrounding a deteriorating patient were sometimes initiated before an escalation occurred when care pathways were predictable to more experienced staff or clearly documented in guidelines for less experienced staff. Staff were aware of time critical elements to escalation such as Sepsis 6 and delivering antibiotics within the ‘golden hour’. Despite the criticality of these tasks, they were prone to interruptions and staff were observed to have competing demands and workload. There were examples where clinical staff were trying to manage two unwell patients simultaneously or, when caring for unwell patients, were interrupted with requests from other patients. To mitigate this, staff worked collaboratively to limit the care deficit for the other ward patients. One clinical staff member described how she had experience of both an outreach organization and one where there was no outreach. She described how, during some patient deterioration episodes, outreach would provide first‐line treatments so she could then manage her other patients. To balance care and resources, some escalations observed involved staff stepping outside of the expected procedure (renal doctor supporting general surgery doctor) to support other clinical areas providing intra‐organizational expertise during deterioration events (see File S4).

## 4 DISCUSSION

The success factors to escalation within this data primarily revolve around the ability of healthcare staff to effectively recognize clinical concerns in varying contexts and how they adapt communication strategies within the escalation process. Of the escalations observed in this study, 51% were triggered by a clinical concern not relating to an elevated EWS; and was a finding supported in both the qualitative and quantitative data. When an escalation was non‐EWS initiated, it predominantly involved symptoms such as bleeding, infection, chest pain and resolved desaturation. When an escalation event was EWS initiated, it predominantly involved low level triggering patients with physiological changes such as those secondary to sepsis, hypotension, reduced conscious level, and hypoxia. To our knowledge, this is the first study, to challenge the concept of escalation homogeneity and identify four escalation phenotypes including Output Focused Escalation, Informative Escalation, General Concern Escalation, and Spontaneous Interaction Escalation. The primary aim of this study was to develop a theoretical understanding of escalation of care in the acute ward setting.

Informative and Spontaneous Interaction Escalations are clinically significant. Informative Escalations were commonly observed, resulting from NEWS2 over‐predicting deterioration, being on the wrong scale and inflexibility within the escalation protocols which dictate clinical actions based on score thresholds. False positive workloads impact the clinical team's ability to deliver care to those patients who would benefit (Forster et al., 2018) and the true number of Informative Escalations may be greater had staff escalated all triggers, which is unlikely as literature suggest only 40% escalation compliance (Connell et al., 2021). Instances of ‘failed escalations’ may be clinician's functioning as a barrier between a false‐positive scores and potentially harmful or costly investigations (Haegdorens et al., 2018). It may be prudent to re‐evaluate the importance of Informative Escalations and assess process enhancements by reducing their frequency (success factors). Doing so would demonstrate improvements in EWS performances and organizational responses to deterioration.

No data exists which differentiates escalation or its communication, but some studies have examined the efficacy between communication modes such as mobile phones or face to face discussions (Gharaveis et al., 2018). Our study data supports that escalation communication is not simply a transfer of information, but collaborative sense making. Maximizing opportunities for Spontaneous Interaction Escalations (success factor), harnessed through environmental (Ede et al., 2021) and system designs, is something which should be explored further with a greater amounts of data. Environmental factors such as layout design, visibility between staff/patients, and accessibility of areas affect the way clinicians interact (Gharaveis et al., 2018). Healthcare designs can promote knowledge exchanges (Lu & Zimring, 2012), therefore a focus should be on maximizing deterioration dialogues (Sujan et al., 2022) when creating healthcare work spaces. Similarly, face to face safety huddles (Franklin et al., 2020), to some extent replicate Spontaneous Interaction Escalations by creating opportunities for inter‐professional communication (Sujan et al., 2022) that generate safety critical tasks such as increasing vital signs frequency, rechecking investigations, or validating clinical concerns.

### 4.1 Strengths and limitations

As the data demonstrate, observing within a clinical area during a patient deterioration episode is sensitive and difficult, which is why no direct patient observations were undertaken during this study. However, this meant that fulfilling all the requirements of the data collection was not feasible for every observation session and explains the data gaps illustrated in the study results. For research purposes without CAG support, identifying unwell patients within the hospital is challenging and this work was undertaken during a period of significant healthcare turbulence where access to clinical areas was significantly restricted. It must be highlighted that not all escalation events were captured during this period, and this data represents a small fraction. Results may not be completely generalisable but do provide valuable insight into escalation recommendations. Also had we observed escalation in different Trusts, data may differ in terms of processes and organizational factors, so results must be viewed with caution. Another influencing factor on this work was the evolving COVID‐19 pandemic which meant the access to some wards were restricted in the early phases of data collection. Finally, whilst every effort was made to reduce the Hawthorne effect and the influence of a researcher being present intrinsically changing behaviour, this may still have biased the data.

### 4.2 Conclusion

A surprising proportion of escalations in this study were initiated by a concern that did not relate to EWS and their true workload is uncertain. It is important to understand non‐triggering escalations to inform the design of systems which support staff who undertake these tasks and improve patient outcomes. Furthermore, there are subtle differences between escalation types and a broad and homogenous definition of escalation is misleading and will not contribute to process improvements. Informative Escalations may be signalling that current escalation policies are too inflexible to support clinical staff fully and warning systems are overpredicting risk. Environmental and system factors may encourage more Spontaneous Interaction Escalations, through well designed clinical spaces, that facilitate deterioration dialogues and improve patient care.

## AUTHOR CONTRIBUTIONS

All authors have agreed on the final version and meet at least one of the following criteria recommended by the ICMJE (http://www. icmje.org/recommendations/): (1) substantial contributions to conception and design, acquisition of data, or analysis and interpretation of data; (2) drafting the article or revising it critically for important intellectual content.

## FUNDING INFORMATION

JE is funded by the National Institute for Health and Care Research [Clinical Doctoral Research Fellowship (NIHR300509)]. The views expressed are those of the author(s) and not necessarily those of the NIHR or the Department of Health and Social Care. The research was supported by the National Institute for Health Research (NIHR) Oxford Biomedical Research Centre (BRC). RE is employed by the National Institute for Health and Care Research.

## CONFLICT OF INTEREST STATEMENT

No conflict of interest has been declared by the author(s) in relations to this study.

### PEER REVIEW

The peer review history for this article is available at https://www.webofscience.com/api/gateway/wos/peer‐review/10.1111/jan.16248.

## Supporting information


File S1.

File S2.

File S3.

File S4.

File S5.

## Data Availability

The data that support the findings of this study are available from the corresponding author upon reasonable request.
