# Supplementary material for: Successfully initiating an escalation of care in acute ward settings—A qualitative observational study
Source: J Adv Nurs. 2024 Jun 27;81(2):887–96. doi: 10.1111/jan.16248 (PMC11729218; doi:10.1111/jan.16248)
Supplement: Supplementary file 1 — File S1. [file JAN-81-887-s005.docx]

Figure 1 Hierarchical Task Analysis (HTA) of Escalation of Care
