# Supplementary material for: Successfully initiating an escalation of care in acute ward settings—A qualitative observational study
Source: J Adv Nurs. 2024 Jun 27;81(2):887–96. doi: 10.1111/jan.16248 (PMC11729218; doi:10.1111/jan.16248)
Supplement: Supplementary file 2 — File S2. [file JAN-81-887-s001.docx]

# Supplementary File. 2 Clinical area descriptions

| Ward Type | Site Descriptor | General Description |
| --- | --- | --- |
| Surgical Assessment Unit Triage | Site A | Front door access for patients, 10 beds which are under direct observation, staffed with ANP, Surgical Junior and Senior Doctors, Care Support Workers |
| Surgical Ward | Site A | Two 23 bedded wards separated by patient gender, 4 side rooms on each ward, staffed with 3 qualified, and 2 HCWs |
| General Surgical Unit | Site B | 40 bedded surgical assessment unit, |
| Emergency Medical Assessment Unit | Site A | Large front door access for patients, situated next to the A+E department, 30 bedded unit, 6 side rooms, staffed with 6-8 qualified and 4 HCWs |
| Accident and Emergency | Site B | 4 resus beds, 35 majors’ beds, paediatrics and adults admissions, assesses around 400 patients per day |
| Surgical Vascular Ward | Site A | 23 bedded vascular wards 4 side rooms, |
| Ambulatory Assessment Unit | Site A | Admission length 5-7 hours, approximately 30 beds |
| Acute Medical Unit | Site B | 52 beds, higher level monitoring ward, |
