# Supplementary material for: Successfully initiating an escalation of care in acute ward settings—A qualitative observational study
Source: J Adv Nurs. 2024 Jun 27;81(2):887–96. doi: 10.1111/jan.16248 (PMC11729218; doi:10.1111/jan.16248)
Supplement: Supplementary file 3 — File S3. [file JAN-81-887-s002.docx]

# Supplementary File. 3 Raw EWS frequency and clinical concern data


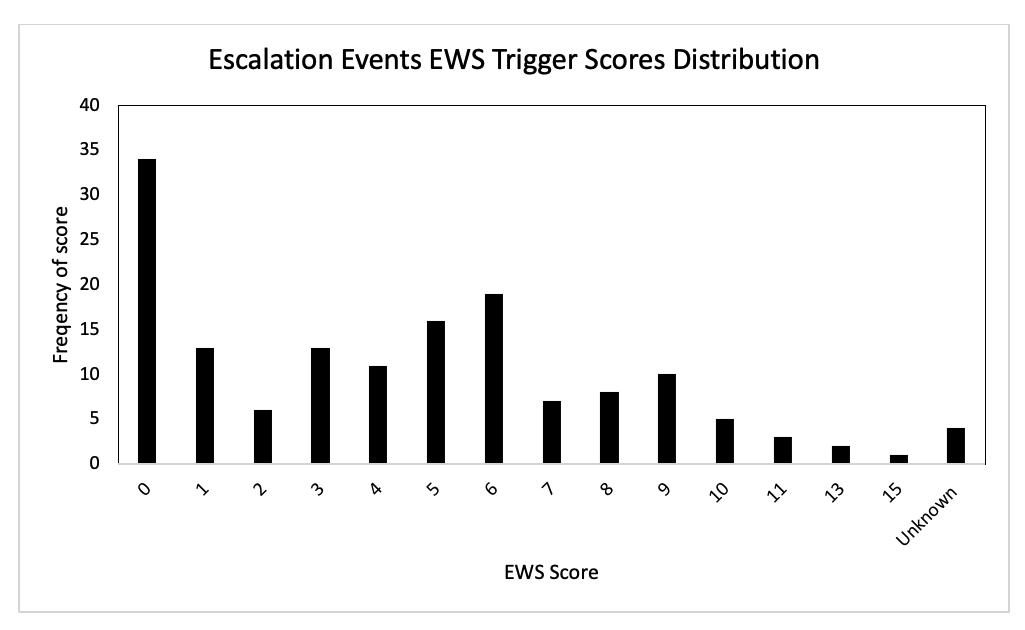


Frequency (n) of score

Figure 1 Escalation Events Trigger Scores Distribution


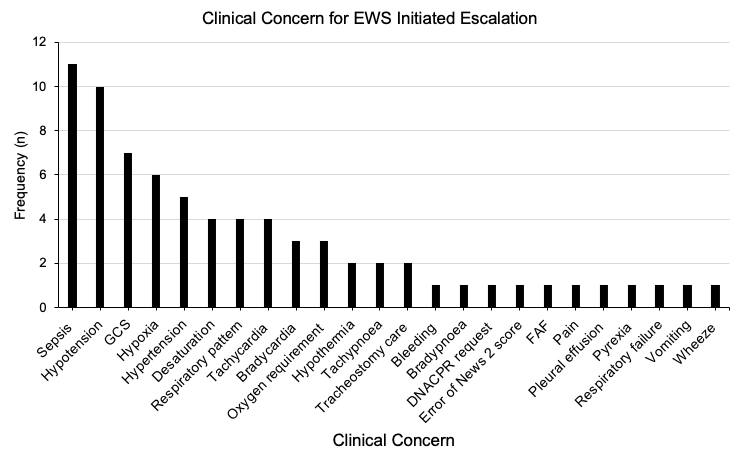
Figure 2 Frequency of Clinical Concerns in EWS Initiated Escalations (n=74)


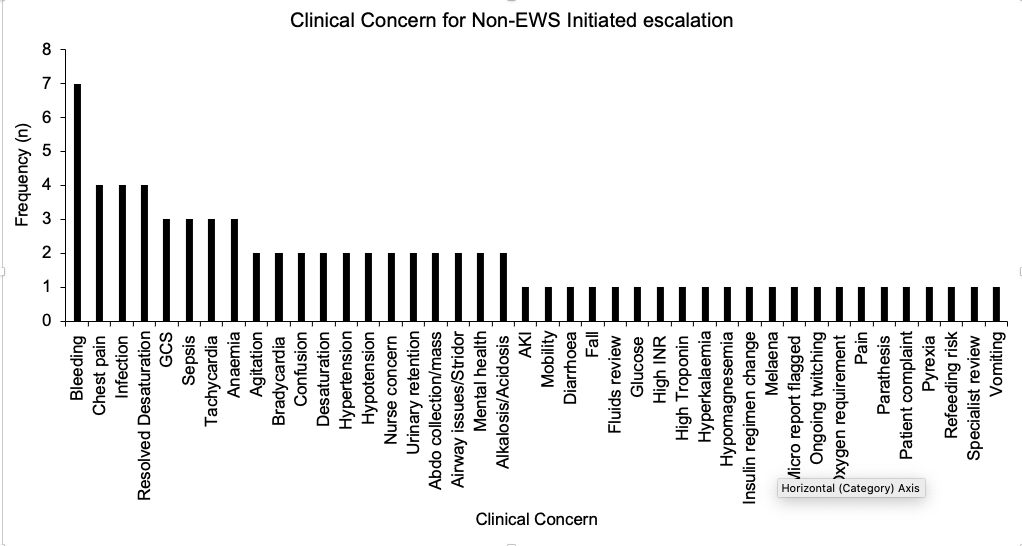


Figure 3 Frequency of Clinical Concerns in Non-EWS Initiated Escalations (n=77)

Table 1 Raw Escalation Concern Data

|  |  |  |  |
| --- | --- | --- | --- |
| Count of Escalation cause | **Initiated by EWS No** | **Initiated by EWS Yes** | **Grand Total** |
| Abdo collection | 1 |  | 1 |
| Abdo mass | 1 |  | 1 |
| Acidosis | 1 |  | 1 |
| Agitation | 2 |  | 2 |
| Airway issues | 1 |  | 1 |
| AKI | 1 |  | 1 |
| Anaemia | 2 |  | 2 |
| Bleeding | 7 | 1 | 8 |
| Blood transfusion | 1 |  | 1 |
| Bradycardia | 2* | 3 | 5 |
| Bradypnea |  | 1 | 1 |
| Chest pain | 4 |  | 4 |
| Confusion | 2 |  | 2 |
| Confusion and mobility | 1 |  | 1 |
| Desaturation | 2* | 4 | 6 |
| Diarrhoea | 1 |  | 1 |
| DNACPR request |  | 1 | 1 |
| Error of News2 score |  | 1 | 1 |
| FAF |  | 1 | 1 |
| Fall | 1 |  | 1 |
| Fluids review | 1 |  | 1 |
| GCS | 3 | 7 | 10 |
| Glucose | 1 |  | 1 |
| High INR | 1 |  | 1 |
| High Troponin | 1 |  | 1 |
| Hyperkalaemia | 1 |  | 1 |
| Hypertension | 2* | 5 | 7 |
| Hypomagnesemia | 1 |  | 1 |
| Hypotension | 2* | 10 | 12 |
| Hypothermia |  | 2 | 2 |
| Hypoxia |  | 6 | 6 |
| Infection | 4 |  | 4 |
| Insulin regimen change | 1 |  | 1 |
| Melaena | 1 |  | 1 |
| Mental health | 2 |  | 2 |
| Micro report flagged | 1 |  | 1 |
| Nurse concern | 2 |  | 2 |
| Ongoing twitching | 1 |  | 1 |
| Oxygen requirement | 1 | 3 | 4 |
| Pain | 1 | 1 | 2 |
| Parathesis | 1 |  | 1 |
| Patient complaint | 1 |  | 1 |
| Pleural effusion |  | 1 | 1 |
| Pyrexia | 1* | 1 | 2 |
| Refeeding risk | 1 |  | 1 |
| Respiratory failure |  | 1 | 1 |
| Respiratory pattern |  | 4 | 4 |
| Sepsis | 3 | 11 | 14 |
| Severe alkalosis | 1 |  | 1 |
| Specialist review | 1 |  | 1 |
| Stridor | 1* |  | 1 |
| Tachycardia | 3* | 4 | 7 |
| Tachypnoea |  | 2 | 2 |
| Tracheostomy care |  | 2 | 2 |
| Urinary retention | 2 |  | 2 |
| Vomiting | 1 | 1 | 2 |
| Wheeze |  | 1 | 1 |
| Resolved Desaturation | 4 |  | 4 |
| Grand Total | **77** | **74** | **151** |

Table 2 Physiological abnormalities that were classified as not a EWS related escalation of care

| Escalation cause | Count | Justification for not being classified as a EWS related escalation of care |
| --- | --- | --- |
| Bradycardia | 2* | Patient did not trigger a 3 in EWS but staff had clinical concerns. |
| Desaturation | 2* | Patient did not trigger a 3 in EWS but staff had clinical concerns. |
| Hypertension | 2* | Patients were re-escalated due to medication requirements. Already previously escalated based on high BP. |
| Stridor | 1* | No triggering score (Spo2, RR). Respiratory effort and audible noise reason for escalation. |
| Hypotension | 2* | Patient did not trigger a 3 in EWS. Staff were more concerned about ongoing rate of PR blood loss which was increasing. |
| Tachycardia | 3* | Patient did not trigger a 3 in EWS but staff were concerned. |
