# Supplementary material for: Successfully initiating an escalation of care in acute ward settings—A qualitative observational study
Source: J Adv Nurs. 2024 Jun 27;81(2):887–96. doi: 10.1111/jan.16248 (PMC11729218; doi:10.1111/jan.16248)
Supplement: Supplementary file 4 — File S4. [file JAN-81-887-s003.docx]

# Supplementary File. 4 Qualitative Themes (Framework Analysis)

Each escalation sub theme documented in the HTA (deterioration detection, deterioration communication and escalation action) was supported by evidence from the observational qualitative data, field notes and researcher reflections/memoirs.

| Escalation Sub task | Key Success Attributes | Excerpt from field notes/ad hoc interviews/researcher reflections |
| --- | --- | --- |
| Deterioration Detection 1 | Pathways to enable **‘Family Escalations’** | *“We recently had a patient that was referred to us by their family, who became progressively more unwell and was admitted to ICU. We have had several examples where patients care has been directly altered due to a family escalation to outreach” Site B/outreach nurse 1*  *“Call for concern a very interesting concept, "at least once a month we have a relative call that initiates a rescue of the patient" Site B/Sepsis Nurse 1* |
|  | Creating opportunities for a broad spectrum of **‘Escalation Actors’** | *‘Housekeeper escalated patient complaint of pain to the nurse in charge.’ Site A/ Esc 28*  *‘Patient desaturated to 82-76 and student escalated to qualified nurse. The replaced oxygen and patient refused to wear oxygen again. They escalated to medical team due difficulty managing. Patient was on a cardiac monitor.’ Site A/ Esc 137*  *‘During one session I was shadowing a surgical ward round. The MDT were reviewing a patient within the side room. During this time an HCA came out of the opposing side rooms and spoke to the nurse in charge. I found out that the HCA had just been mobilising this patient with the physiotherapy team which she had done previously. She was concerned because the patient was notably short of breath of exertion, more so than previous rehabilitation sessions. The nurse in charge suggested that this patient been seen next and diverted the ward round to this patient. This started several interventions such as a chest x-ray and full medical review’ Site A/Observation Session 3* |
|  | Understanding the advantages and limitations of **‘Early Warning Scores’** | *“It is easier when the EWS trigger but I can still escalate when they don't. I think the junior staff rely more on the EWS to direct escalation” Site A/Nurse 1*  *‘Medics don't change the scale for COPD patients. Just seen a patient triggering 4, background of COPD and still on scale 1’ Site A/ Nurse 3*  *‘Patient admitted having had surgical procedure few days previously, history of rigors at home. Was admitted and given ABx. Patient was really keen to go home. Was going to be discharged but nurses not happy given history despite not triggering. Patient kept in overnight developed sepsis and became unwell.’ Site A/ Esc 27*  *“COVID patients don't initially score highly on NEWS but have huge potential to become more unwell.” Site A/Nurse 2.*  *‘When clinical staff are concerned about patients, they increase monitoring from ad hoc monitoring to continuous monitoring. This is for both prevention and efficiency. Prevention to allow early detection of changes whilst reducing workload of increased vital signs measurements required” Site A/ Observation Session 11* |
|  | Understanding the **‘Complexity and Nuance of Deterioration’** | *‘Went to see a patient who had a sepsis flag, but discussion with docs suggest no infective concern. Contacted later in the afternoon to say patient had dropped blood pressure, and now on IVAbx.’ Site A/Observation Session 10*  *‘Key escalation was the concern for a patient that has ongoing diarrhoea following a Covid injection and rising inflammatory markers. Although she looks well, team want to exclude abdominal pathology following Whipples’ Observation Session 1*  *“Patients who come in have physiology normalisation with fluids and oxygen then don't meet sepsis criteria.” Site B/Sepsis Nurse 1*  *‘Data to show that most referrals are based around nurse concern.’ Site B/ Observation Session 7* |
|  | Technology that promotes the **‘Visibility of Deterioration’** | *‘Electronic notice boards feature heavily in these escalation events. This generates and accessibility of information.’ Observation Session 7*  *“Patient had already been identified earlier in the day by NEWS Review” Site B/Esc 25*  *‘Today has 15 sepsis flags hospital wide’ Site A/Observation Session 10* |
|  | **‘Technology Adaptability and Usability’** to meet users’ needs | *‘Discussion with consultant about surgical leaks which occur on day 3 to 5. Has an alert for CRP on patients operated on earlier in the week. This gives an indication about leak prior to trigger. Young patients can compensate a leak, but older frailer patients can’t as well and are much sicker’ Site A/ Surgical Consultant* |
| Escalation Communication 2 | Creating a ‘**Social Currency’** to facilitate escalation | *‘Social relationships with patients, relatives and medical team generates a form of ‘social currency’ element to escalating care. This can smooth the course when escalating to people known to each other. This was particularly evident with the Outreach Teams’ Site B/ Observation Session 13*  *‘One ward had 38 patients and 9 consultants sharing patient care plus take consultants. Finding the right person to escalate to and then contacting them is very challenging and time consuming’. Site A/ Observation Session 12*  *‘Patient admitted for a week following biopsy of sigmoid. Had 3litres of fluid. For resus. VBG done about an hour ago by ward staff. Outreach knows the nurse referring and she is very experienced.’ Site B/ Esc 29* |
|  | Allowing for **‘Multiple Modes of Communication’** | *“Surgical Registrar phoned in from home and asked junior doctor to give stat dose of Gent to a patient. Not sure what triggered this interaction, but clearly the doctor accessed new patient information. Patient had this prescribed’ Site A/ Esc 12*  *‘Staff use multiple modes of communication. Frequently referring to handover emails on mobile devices.’ Site A/Observation Session 1* |
|  | Developing the skills in forming a convincing **‘Deterioration Narrative’** | *"If you don't use the right language to escalate then it may not be taken seriously" Previous call today, complete jumble. Advised to use SBAR to organise call” Site B/Observation Session 6* |
| Escalation Action 3 | **‘Predictable Care Pathways’** such as Sepsis 6 | *‘Patient came in from a nursing home. Looks very unwell. Currently being escalated to nurse in charge and then this being escalated to sepsis nurse. Patient is not for resus. Being reviewed by Medical reg…... On discussion the nurse is very conscious of the golden hour for ABx delivery to meet the Sepsis 6 criteria.’ Site A/ Observation Session 11*  *“Patient is clearly unwell. Concurrent tasks being completed. Manual bp monitor at bedside as bp low. VBG completed. Nurse in charge at bedside as well. Trying to get access in. On a continuous monitor. Noted lactate 2.6 with high potassium. Nurse recognised that patient was septic based on observations. Knew sepsis 6 would be initiated and that antibiotics would be needed. Therefore, escalated to medics straight away to ensure this was done. History and observations were directing to sepsis. Uses a sepsis 6 crib sheet on ID cards.” Site A/Esc 37* |
|  | **‘Mitigating the impact to the Wider Ward’** Population | *“Nurse came from MET system. Knew the plan would be organised, because the MET team would look after sick patient which meant that you could look after case load” Site A/Nurse 3*  *‘Despite nurse looking after sick patient, she also then had to help two patients to the toilet. Whilst she did this the nurse in charge seamlessly took over sick patients care’ Site A/ Observation 11*  *‘The same nurse has two patients triggering. Nurse actively encouraging family to participate in care’ Site A/ Observation 16* |
|  | **‘Collaborative Sense Making’** to identify key escalation tasks | *‘Patient who was seen overnight and re-referred. Doctor letting outreach know patient still unwell and now struggling. Tried nebs and not improving. SHO referred. Outreach review prompted a DNACPR discussion between team and family.’ Site B/ Esc 148*  *“Patient had already been identified earlier in the day by NEWS Review. Reassuring staff that she has a reason for high trigger, and this is being treated with diuretics and known to have pleural effusions.” Site B/ Esc 25*  *“..when we did visit the outreach nurse was concerned that she was still doing neuro observations with a patient who is likely palliation. Outreach contacted team to ask to review and reduce unnecessary interventions” Site B/ Esc26*  *‘Observing Safety Huddle this am on Neurosciences ward which centred around the large ward whiteboard. Patients who were a clinical concern were discussed. Some of these patients were not triggering or had high early warning scores. The actions that staff generated from these meetings may not have been addressed had this discussion not occurred’ Site A/ Esc 141* |
|  | **‘Utilising Unexpected Resources’** | *‘Asked to see patient by FY1 (day team to the night team handover). Whilst reviewing the nurse requests review of urine for haematuria…. SHO from Urology helped review concerning patients’ Site A/ Observation Session 3* |
