# Supplementary material for: Successfully initiating an escalation of care in acute ward settings—A qualitative observational study
Source: J Adv Nurs. 2024 Jun 27;81(2):887–96. doi: 10.1111/jan.16248 (PMC11729218; doi:10.1111/jan.16248)
Supplement: Supplementary file 5 — File S5. [file JAN-81-887-s004.docx]

# Supplementary File. 5 SPSS Outputs for Clopper-Pearson 95% Confidence Intervals

| **Escalation Phenotypes** | | | | | |
| --- | --- | --- | --- | --- | --- |
|  | | Frequency | Percent | Valid Percent | Cumulative Percent |
| Valid | Informative | 49 | 5.0 | 35.8 | 35.8 |
|  | General Concern | 26 | 2.7 | 19.0 | 54.7 |
|  | Outcome focused | 57 | 5.8 | 41.6 | 96.4 |
|  | Spontaneous Interaction | 5 | .5 | 3.6 | 100.0 |
|  | Total | 137 | 14.0 | 100.0 |  |

| **Confidence Interval Summary** | | | | |
| --- | --- | --- | --- | --- |
| Confidence Interval Type | Parameter | Estimate | 95.0% Confidence Interval | |
|  |  |  | Lower | Upper |
| One-Sample Binomial Success Rate (Clopper-Pearson) | Probability(Escalation=Informative). | .358 | .278 | .444 |

| **Confidence Interval Summary** | | | | |
| --- | --- | --- | --- | --- |
| Confidence Interval Type | Parameter | Estimate | 95.0% Confidence Interval | |
|  |  |  | Lower | Upper |
| One-Sample Binomial Success Rate (Clopper-Pearson) | Probability(Escalation=General Concern). | .190 | .128 | .266 |

| **Confidence Interval Summary** | | | | |
| --- | --- | --- | --- | --- |
| Confidence Interval Type | Parameter | Estimate | 95.0% Confidence Interval | |
|  |  |  | Lower | Upper |
| One-Sample Binomial Success Rate (Clopper-Pearson) | Probability(Escalation=Outcome Focused). | .416 | .333 | .503 |

| **Confidence Interval Summary** | | | | |
| --- | --- | --- | --- | --- |
| Confidence Interval Type | Parameter | Estimate | 95.0% Confidence Interval | |
|  |  |  | Lower | Upper |
| One-Sample Binomial Success Rate (Clopper-Pearson) | Probability(Escalation=Spontaneous Interaction). | .036 | .012 | .083 |
